# Draft Genome Sequence of the Patulin-Producing Fungus Paecilomyces niveus Strain CO7

**DOI:** 10.1128/genomeA.00556-18

**Published:** 2018-06-21

**Authors:** Megan N. Biango-Daniels, Tristan W. Wang, Kathie T. Hodge

**Affiliations:** a Plant Pathology and Plant-Microbe Biology Section, School of Integrative Plant Science, Cornell University, Ithaca, New York, USA

## Abstract

Paecilomyces niveus is an extremotolerant fungus with surprising powers to survive high temperatures and infect apples and aphids. These abilities make it a formidable enemy in food and agricultural environments. In addition, it produces patulin, the most significant mycotoxin in apples.

## GENOME ANNOUNCEMENT

Paecilomyces niveus Stolk & Samson (Byssochlamys nivea Westling) (Ascomycota, Eurotiomycetes, Eurotiales, Thermoascaceae) produces heat-resistant ascospores, is capable of growth in low-oxygen environments, and produces numerous mycotoxins (1). Members of the genus are widespread in soils and are often associated with spoilage of thermally processed or pasteurized foods (1). Paecilomyces niveus has been found to produce mycophenolic acid, byssochlamysol, byssochlamic acid, and patulin; the latter mycotoxin is associated with apples and is restricted by the FDA to 50 ppb in apple products (1–4). Paecilomyces niveus has been implicated in spoilage of packaged fruit products (1, 3, 5), was recently demonstrated to cause the postharvest apple disease *Paecilomyces* rot (6), and can infect aphids (7). Sequencing of the P. niveus genome allows further investigation into the mechanisms behind its pathogenicity and growth under extreme conditions. For example, the closest fully sequenced relative of P. niveus, Paecilomyces variotii (Byssochlamys spectabilis), degrades formaldehyde (8).

Paecilomyces niveus (Byssochlamys nivea) strain CO7 (Cornell Orchards number 7) was isolated from a decaying apple at Cornell Orchards in Ithaca, NY, in 2014. Its genome was sequenced at the Cornell University Biotechnology Resource Center (BRC) via a whole-genome shotgun strategy using Illumina MiSeq, applying a paired-end 2 × 250-bp approach. Jellyfish (version 2.2.3) was used with a k-mer size of 31 based on the adapter-trimmed file to estimate the genome size as 44.0 Mb (9, 10). Assembly was performed with IDBA-UD (version 1.1.1), scaffolds less than 1 kb in length were removed, and the gene content completeness of the final assembly was assessed with BUSCO (version 3.0.2) based on the eurotiomycetes_odb9 gene set (11, 12).

In total, 9,158,889 paired-end reads were assembled into 586 large contigs (*N*_50_, 185,295 bp; *N*_90_, 43,184 bp; *L*_50_, 60 contigs; *L*_90_, 107 contigs; G+C content, 47.11%). This draft genome included a total sequence length of 36,018,796 bp with a maximum contig length of 705,283 bp. Genome annotation of the inferred scaffolds was performed via the Joint Genome Institute (JGI) annotation pipeline (13), resulting in 10,584 open reading frames. The average gene density was one gene per 1,480 kb, and on average, each gene had 3.57 exons. The average exon size was 415 bp. There were approximately 27,201 introns; the average intron size was 93 bp, and the average number of introns per open reading frame was 2.57.

BLASTp was used to search for putative secondary metabolite genes responsible for production of the mycotoxin patulin, which has been shown to enhance virulence of the apple pathogen Penicillium expansum (14). The program predicted a cluster of 15 putative genes in P. niveus responsible for patulin production, all of which were syntenic with the related patulin-producing species Aspergillus clavatus (15).

### Accession number(s).

This whole-genome shotgun project for P. niveus CO7 (NRRL 66824) has been deposited at DDBJ/ENA/GenBank under the accession number QEIL00000000. The version described in this paper is version QEIL01000000.
